# Autophagy Intertwines with Different Diseases—Recent Strategies for Therapeutic Approaches

**DOI:** 10.3390/diseases7010015

**Published:** 2019-02-01

**Authors:** Janani Ramesh, Larance Ronsard, Anthony Gao, Bhuvarahamurthy Venugopal

**Affiliations:** 1 Department of Medical Biochemistry, Dr. A.L.M. Post Graduate Institute of Basic Medical Sciences, University of Madras, Chennai 600113, India; rjjananiramesh95@gmail.com or jramesh1@bwh.harvard.edu; 2 Renal Division, Brigham and Women’s Hospital, Harvard Medical School, Boston, MA 02115, USA; Anthony.Gao@oberlin.edu; 3 The Ragon Institute of Massachusetts General Hospital, The Massachusetts Institute of Technology and Harvard University, 400 Technology Square, Cambridge, MA 02140, USA; Lronsard@mgh.harvard.edu

**Keywords:** autophagy, cancer, neurodegenerative disorder, lysosomal disorder, renal disorder, inflammation, HIV, drug toxicity

## Abstract

Autophagy is a regular and substantial “clear-out process” that occurs within the cell and that gets rid of debris that accumulates in membrane-enclosed vacuoles by using enzyme-rich lysosomes, which are filled with acids that degrade the contents of the vacuoles. This machinery is well-connected with many prevalent diseases, including cancer, HIV, and Parkinson’s disease. Considering that autophagy is well-known for its significant connections with a number of well-known fatal diseases, a thorough knowledge of the current findings in the field is essential in developing therapies to control the progression rate of diseases. Thus, this review summarizes the critical events comprising autophagy in the cellular system and the significance of its key molecules in manifesting this pathway in various diseases for down- or upregulation. We collectively reviewed the role of autophagy in various diseases, mainly neurodegenerative diseases, cancer, inflammatory diseases, and renal disorders. Here, some collective reports on autophagy showed that this process might serve as a dual performer: either protector or contributor to certain diseases. The aim of this review is to help researchers to understand the role of autophagy-regulating genes encoding functional open reading frames (ORFs) and its connection with diseases, which will eventually drive better understanding of both the progression and suppression of different diseases at various stages. This review also focuses on certain novel therapeutic strategies which have been published in the recent years based on targeting autophagy key proteins and its interconnecting signaling cascades.

## 1. Introduction

Autophagy is a well-studied adoptive intracellular homeostasis process through which impaired cellular materials, senescent organelles, and biological surplus macromolecules are eliminated with the help of lysosomal hydrolytic enzymes [1,2,3,4]. Various reports on cellular mechanisms have shown that different types of autophagy pathways were found within the cells, which include macro-autophagy, micro-autophagy, and chaperone-mediated autophagy [1,5]. The process of autophagy has been well-known since it was discovered by Christian de Duve, who observed the degradation of mitochondria and other intracellular structures within the lysosomes of rat livers perfused with glucagon [6]. However, the fundamental concepts of autophagy were recognized only after 1995 from the findings of Yoshinori Ohsumi, whose significant contribution earned him a Nobel prize in physiology or medicine in 2017. This review solely aims to highlight the gradual and rapid advances in the autophagy field in various years and intervals. Here, we will discuss how autophagy dysregulation interlinks with several diseases, namely neurodegenerative diseases, Lysosomal Storage Diseases (LSD), cancer, renal disorders, inflammatory diseases, and HIV. This review will provide cellular and molecular information of autophagy in various diseases for targeting and developing novel strategies against diseases.

## 2. Overview of Autophagy for Intracellular Homeostasis

**Figure 1:**
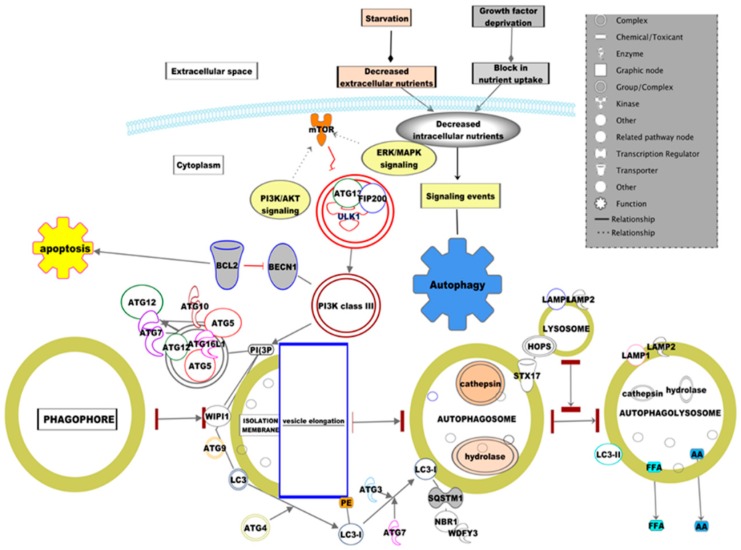
The autophagy-A highly conservative cascaded regulatory mechanism upholds the intracellular homeostasis of the biological system. Five steps are involved in the autophagy signaling cascades: 1. Nucleation of the phagophore; 2. expansion of the phagophore; 3. formation of the autophagosome; 4. fusion of the autophagosome–lysosome; and 5. degradation and efflux of the cargos are illustrated in the pathway. Using a build tool in the pathway developer of IPA (Ingenuity Pathway Analysis), the Autophagy–Canonical pathway was developed.

**Figure 2:**
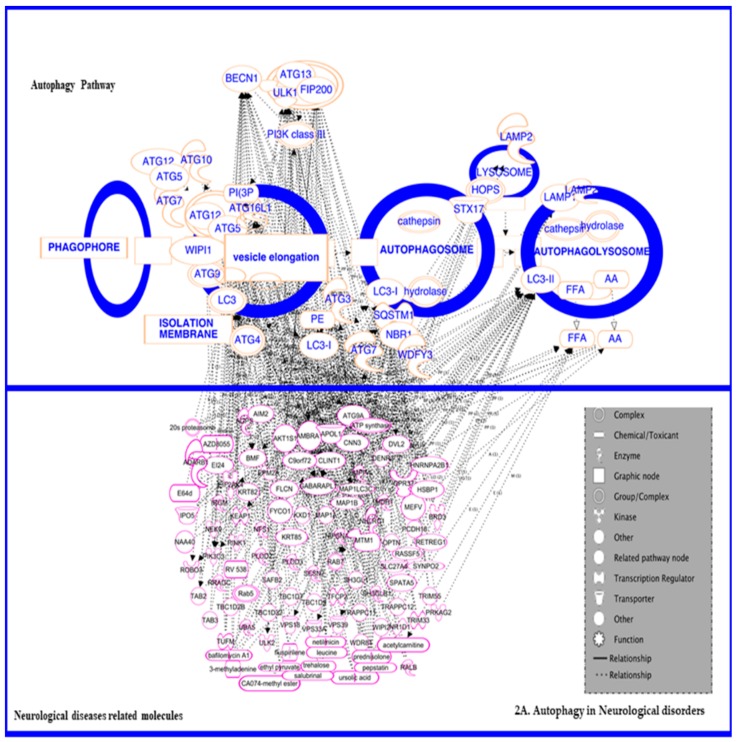

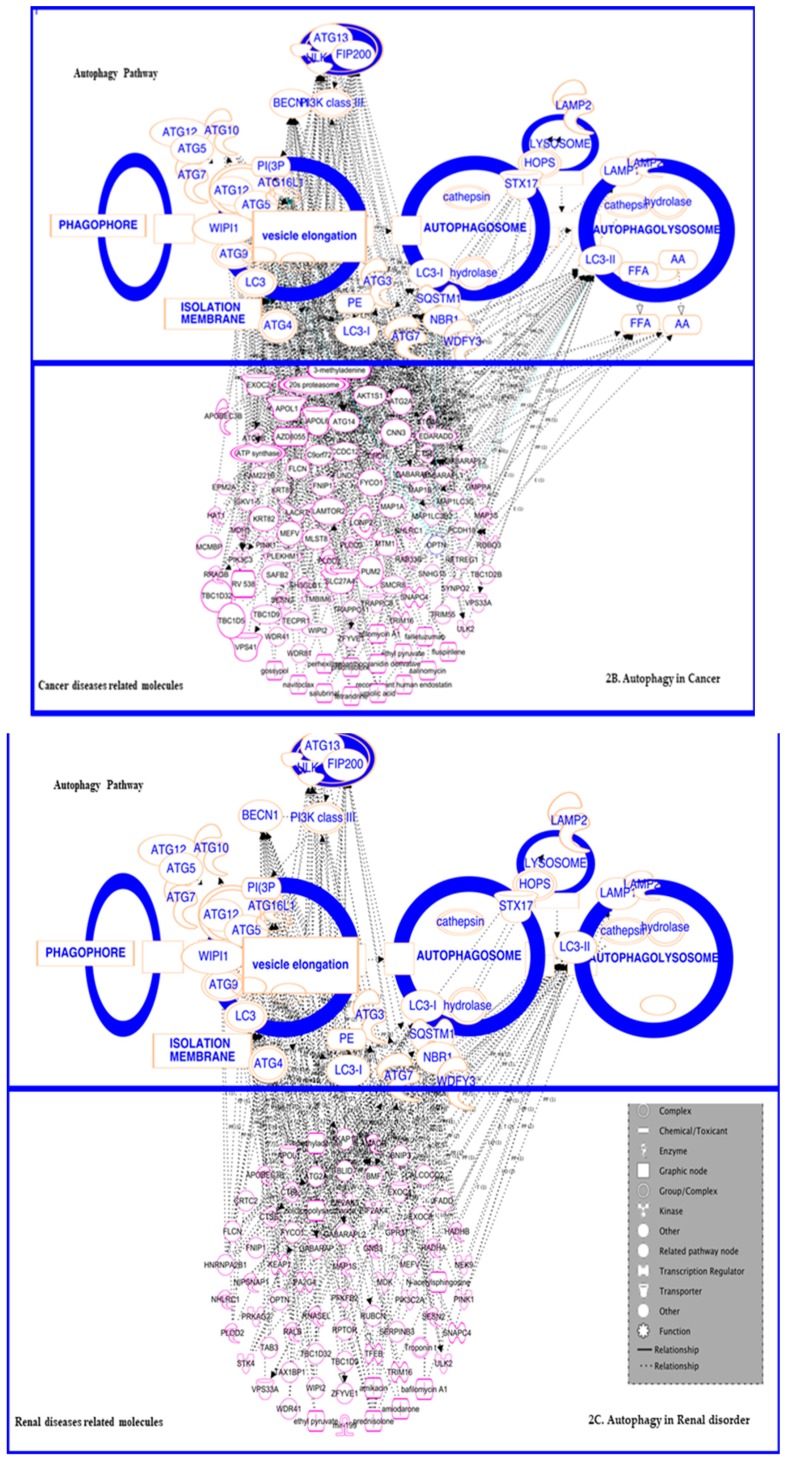

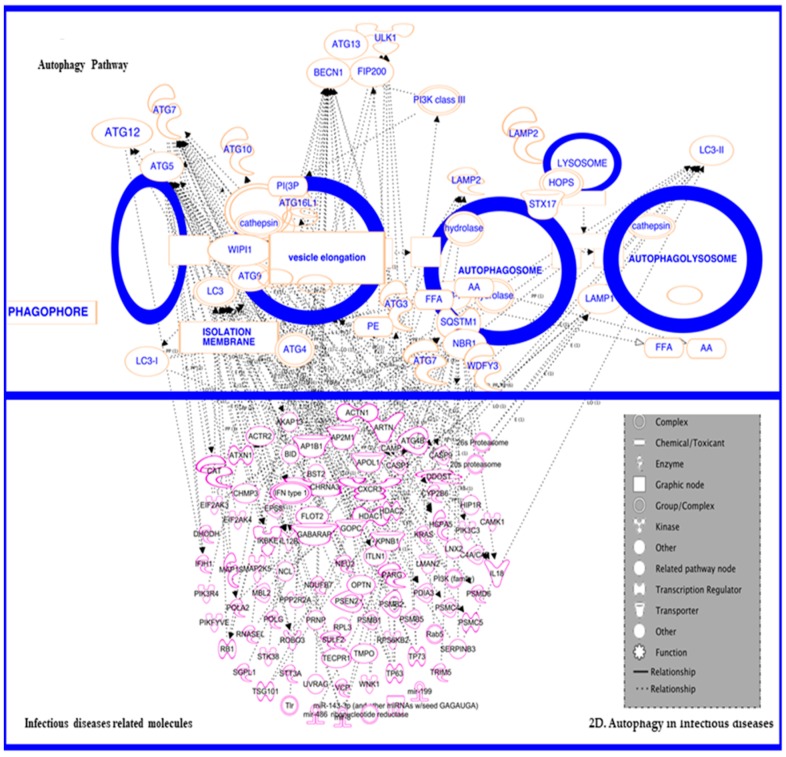
The interconnection of autophagy-related proteins in (**A**) neurological diseases, (**B**) cancer, (**C**) renal disease, and (**D**) infectious diseases. All these figures represent the critical events and key molecules which are connected between autophagy and diseases. In panel 2A, the autophagy proteins, namely LC3-II, BECN1, ATG 13 complex, LAMP2, ATG10, ATG12, ATG7, and ATG5, have been predicted to interlink with neurological diseases-related molecules, such as AMBRA, GABARAPL, VPS18, VPS39, VPS33A, OPTN, KEAP1, Rab5, TRIM55, TRIM33, AKT1S1, APOL1, AIM2, MAP1B, and others. In panel 2B, autophagy proteins, namely LC3-II, BECN1, ATG 13 complex, LAMP2, ATG10, ATG12, ATG7, and ATG5, have been predicted to interlink with cancer-related molecules, such as APOBEC3B, VPS41, TRIM55, TRIM16, AKT1S1, APOL1, APOL6, ATG14, CNN3, MAP1B, ROBO3, SYNPO2, PUM2, SAFB2, and others. In panel 2C, autophagy proteins, namely LC3-II, BECN1, ATG 13 complex, LAMP2, ATG10, ATG12, ATG7, and ATG5, have been predicted to interlink with renal disease-related molecules, such as APOBEC3B, TAB3, VPS33A, TRIM16, WDR41, WIP12, STK4, TFEB, SNAPC4, PIK3C2A, PINK1, MDK, OPTN, KEAP1, FNIP-1, and others. In panel 2D, autophagy proteins, namely LC3-II, BECN1, ATG 13 complex, LAMP2, ATG10, ATG12, ATG7, and ATG5, have been predicted to interlinks with infectious disease-related molecules, such as TLR, UVRAG, WNK1, TP63, TP73, TRIM5, SERPINB3, Rab5, RPL3, IL-18, CXCR3, APOL1, ACTN1, ACTR2, BST2, BID, ATXN1, CAT, CHMP3, NCL, ITLN1, LNX2, PARG, and others. Using a build tool in the pathway developer of IPA (Ingenuity Pathway Analysis), the Autophagy–Canonical pathway was developed; then, the grow tool was used to connect the autophagy-related proteins to the neurological diseases, cancer, renal diseases, and infectious diseases pathways.

The autophagy process requires several consecutive steps to degrade the cargos of senescent organelles captured and the biological surplus macromolecules in the cytosol (Figure 1). The first step in this process is the stimulation of the autophagy signal and formation of autophagosome precursor molecules. The next step is the formation of a complete of double-membrane rings called an “autophagosome” which fuses and docks with lysosomes resulting in “auto-phagolysosome”. Autophagy exploits the above-discussed phases to degrade the targeted or random cargoes. Then, the degraded cargoes generate amino acids and fatty acids which get transported back into the cytoplasmic pool from the Endoplasmic Reticulum (ER) and Golgi. In yeast, more than 30 autophagy-related (Atg) genes have been identified, and most of those are conserved among higher eukaryotes [7]. In the induction phase of autophagy, an isolated membrane is formed and expanded by a dynamic family of proteins characterized as Atg (Atg 12, 7, and 10 and Atg 5/12/16 conjugated) [8,9]. Like Atg proteins, the LC3 complex protein (exists on cell surface in two forms: LC3-I and LC3-II, known for their ubiquitin-like modifier) is also well-known for its contribution in autophagosome formation [10]. The LC3 dispensation is critical for the expansion and elongation of the budding curvatures. The molecular events of autophagy in cells are stimulated by various factors, either by external or internal stress response, including cancer, exercise, caloric restriction, denervation, and nutrient withdrawal. A study has been reported on the low-nutrient status of cells, which spotted a decreased expression (inactivation) of target of rapamycin (TOR) kinase (stress sensor) which lead to Atg13 hypophosphorylation. A complex of genes (Ulk1 gene/Atg1 gene) interacts with the TOR kinase inactivation mediated hypophosphorylation of Atg13 and Atg17 proteins to regulate the importation of lipids through the Atg9 protein. Atg9, a transmembrane protein, has been reported as a key player in “auto-phagophore” formation. The protein complex Vps34/Beclin1 (mammalian orthologue of Atg6 which positively regulates autophagy through the activation of Vps34) conjugates with the Atg5–Atg12 complex which in turn forms a complex with Atg16L by phosphoinositol (PI) conversion to (phosphoinositol 3 phosphate) PI3P. This complex interactome results in the multimerization of the phagophore and formation of budding curvatures. The forthcoming section discusses the sequential description of how this intracellular homeostasis mechanism interlinks with various diseases (Figure 2).

## 3. Role of Autophagy in Diseases

### 3.1. In Neurodegenerative Diseases

Numerous reports have shown that autophagy and some major autophagy-linked proteins play a role in neurological disorders (Figure 2A) such as Parkinson’s disease, Huntington disease, Alzheimer’s disease, prion disease, and transmissible spongiform encephalopathies. Studies on neurodegenerative disorders have reported massively higher levels of autophagosomes in neurodegenerative disease patients, which signifies the importance of autophagy in these cases. Autophagy is very pivotal for performing housekeeping activities in neuronal cells and preventing the buildup of misfolded aggregates in neurons, which can lead to neuronal dysfunction such as Aβ in Alzheimer disease [11], PRNPSC in prion diseases [12], SNCA in Parkinson disease [13,14], and mSOD1 in familial Amyotrophic Lateral Sclerosis (fALS) [15]. Previously reported studies on knockout mouse models lacking important autophagy-related proteins, namely those produced by the Atg5 [16] or Atg7 gene [17], demonstrated severe neurodegeneration in the central nervous system during the absence of autophagy.

In general, the system of ubiquitin–proteasomes is well-known for the clearance of soluble and misfolded proteins in the cytosol [18]. However, a defective ubiquitin–proteasome system leads to large buildup of proteins. This protein buildup may lead to the formation of oligomers of toxins or large aggregates, which tends to affect cellular function and cause neurodegeneration in neurons. Certain diseases like Huntington’s disease, Alzheimer’s, Prion, and Parkinson’s are caused by the accretion of certain protein aggregates in the brain due to a flawed degradation system [19,20]. These aggregates come from the stimulation of mutations in specific genes related to these diseases. In Huntington’s disease, the poly glutamine encoding gene is mutated [21] while in Alzheimer’s disease, mutations in the amyloid precursor protein or the microtubule-associated protein tau [22] result in disease conditions. 

Interestingly, in patients’ cells suffering from these age-related diseases, the accumulation of autophagy vacuoles was found to contain ubiquitinated aggregates of the disease-related proteins. However, it is unexpected due to the functional independency of the cytosolic ubiquitin–proteasome degradation pathway and lysosome-related autophagic process. Observations by [17] Komatsu et al. showed that the conditional knockout of autophagy-related genes lead to neurodegeneration with ubiquitin-positive pathology. Moreover, [23] Pandey et al. recently showed that autophagy acts as a compensatory degradation system when the ubiquitin–proteasome system is impaired in Drosophila melanogaster [13]. Consistent with these observations, Bjorkoy et al. [24] noticed that ubiquitinated cytosolic protein aggregates can be selectively degraded by autophagy and interact with Atg8 protein. This process involves the p62 protein, which is also called sequestosome 1 (SQSTM1). Hence in neurodegenerative diseases, these ubiquitinated protein aggregates are highly associated with the process of autophagy, as evidenced by the interaction between p62 and human Atg8 paralogs [25,26]. 

Autophagosomes accumulate in the brains of patients affected by neurodegeneration as reviewed by References [25,26], and this paves a way to explore autophagy’s contribution to the pathogenesis of neuro-disorders. The stimulation of autophagy by several drugs diminishes high levels of soluble and aggregated forms of the mutant huntingtin protein in Huntington’s disease, mutated α-synuclein proteins in spinocerebellar ataxia, and the mutant tau in Alzheimer’s disease. Autophagy reduces the cellular toxicity in neuronal cell lines, and it is involved in the clearance of mutant protein aggregation-mediated neurotoxicity as reported in mouse and Drosophila models. In drosophila, neuroprotection was observed due to the autophagy-aided clearance of the aggregated proteins or toxins [26]. The overexpression of Histone deacetylase-6 (HDAC-6) was reported to induce autophagy. Pandey et al. showed that ubiquitin–proteasome system impairment in spinal-bulbar muscular dystrophy was reduced by autophagy in the fly model. As per several studies, autophagy was reported as a process leading to muscle wasting and atrophy in several muscular diseases, namely dystrophies, myopathies, and neurogenerative disorders [15,27,28]. Interestingly, a study of the skeletal muscle with the absence of Atg7 resulted in no preservation of the muscle mass on the catabolic state, and during the denervation state, the muscle loss worsened, showing that autophagy is essential for the conservation of muscle mass [29]. Moreover, a recent study by Eva Pigna (2018) highlights the importance of autophagy in regulation by reporting the increased expression levels of the autophagy markers such as LC3b and p62 during denervation, showing that autophagy does not improve muscle fiber atrophy [30]. 

In C. elegans, the knockdown or knockout of the ATG gene increases the formation of aggregate and toxicity of poly Q expansion proteins [31]. It was also reported that in patients of neurodegenerative disease, autophagy may reach a saturation point in which its capacity to degrade the mutant aggregate-prone proteins is exceeded or those concurrent defects may occur in the autophagy pathway. The heterozygous mutant mouse p38-alpha (Mapk14) gene (knockout) in mouse neurons increases autophagy in mouse brains that involves the transgenic mutant human APP protein in mouse. The p38α MAPK deficiency in neuronal cells attenuate amyloid pathology in Alzheimer’s disease via lysosomal degradation of BACE1 in mouse models and also in-vitro models [32]. The formation of peripheral myelin protein 22 aggregates is hindered by the enhancement of autophagy and expression of cytoplasmic chaperones. The interference of human ARF6 mRNA by siRNA affects the formation of autophagosomes in Hela cells. Arf6 promotes autophagosome formation via the effects on phosphatidylinositol 4,5-bisphosphate and phospholipase D. Yoshimura et al. (2006) [33] showed that acquired defects in autophagosome formation may result from two processes: 1. The sequestration of autophagy proteins in aggregates which are formed by mutant proteins and 2. the age-related decline that occurs in Beclin 1 and 3 that are potentially considered as autophagy protein expression in human brain and other as-of-yet unidentified factors.

#### Autophagy in Neurodegenerative Protection

Studies in model organisms show that autophagy defends against various neurodegenerative diseases and the autophagosomes aggregation predominantly signify that autophagy activation is beneficial in the case of Alzheimer’s disease, which is the consequence of a defect in autophagosome maturation [25,34,35]. Autophagy’s role in neuroprotection is not well-established in patients; however, there are few reported studies available for in vitro and in mouse models. Some trials have shown that autophagy-stimulatory agents are beneficial in reducing neurotoxicity (by reducing mutant-aggregated proteins using autophagy-stimulatory agents); they are used to treat other diseases. As reported by Rubinsztein et al. [25], analogs of rapamycin were tested in phase II oncology trials and showcased that analogs of rapamycin have the ability to defend against neurodegeneration in Drosophila and mouse poly Q disease models. Interestingly, lithium chloride, a drug used for the treatment of bipolar disorder, induces autophagy by decreasing the Inositol trisphosphate (IP_3_) levels and accelerating the aggregate-prone protein clearances [25]. Sarkar et al. [25] specifies the importance of the enhancers of rapamycin (SMERs) which are involved in the protein clearances of aggregates [35,36]. These aggregates are reported to be toxic to the cells, especially during the post-mitotic cell stage in neural cells [9,37]. 

The various knockout studies on neuronal cells showed massive deposits of protein aggregates (ubiquitin-related proteins) upon silencing specific autophagy genes, indicating the role of autophagy in protecting neurodegenerative disorders [17,38]. Certain studies showcased the involvement of mutations in vesicular endosome-related proteins (known for auto-phagolysosome formation), named as ESCRT III subunits and CHMP2B or mSnf7-2, which are associated with two neurodegenerative diseases, namely frontotemporal dementia and amyotrophic lateral sclerosis, respectively. Both diseases are characterized by abnormal ubiquitin-related proteins aggregate in affected neurons. Likewise, in-vitro cell line models and fruit flies resulted in decreased autophagy degradation and increased levels of ubiquitin positive aggregates that have been shown to increase neurodegeneration upon the depletion of CHMP2B or mSnf7-2 [39]. Recent studies revealed the role of presynaptic endocytic proteins in the formation and transport of autophagosomes and thus indicated an interlink between autophagy and neuronal proteostasis [40,41].

### 3.2. In Lysosomal Storage Diseases (LSDs)

Lysosomal storage disorders (LSDs) make up a family of disorders caused by inherent gene mutations which perturb lysosomal homeostasis and are typified by the progressive accretion of undigested macromolecules residing in the cell. The lysosome is an important organelle for the autophagy pathway, where it fuses with autophagosomes and degrades autophagic cargoes. Thus, dysfunction in lysosomes in LSDs impacts the autophagy pathway [42]. In fact, in most LSDs, there is an impaired autophagic flux which leads to the dysfunction of lysosomes, resulting in the dysfunction of the autophagosome–lysosome fusion process of autophagy, which in turn results in the accumulation of autophagy substrates like poly-ubiquitinated proteins, damaged mitochondria, and SQSTM1/p62 proteins [43,44]. One rare form of LSD known as Danon disease is caused by a null mutation in the LAMP2 gene (codes for integral lysosomal membrane proteins that also plays a role in CMA) rather than a lysosomal hydrolase encoding gene [45]. LAMP-1 (lysosomal membrane protein) co-localization has been shown to be reduced significantly by the diminished levels of the autophagosome marker LC3. This indicates that the fusions of lysosomal compartments with autophagosomes were impaired [46].

#### 3.2.1. In Mucolipidosis Type IV (MLIV)

It has been proven that mucolipidosis type IV (MLIV), a neurodegenerative lysosomal storage disease, is associated with impaired Chaperone Mediated Autophagy (CMA) [47]. It is marked by mutations in the MCOLN1 gene, which codes for the transient receptor potential mucolipin I (TRPML1), a cation channel localized to lysosomes and late endosomes. Loss of TRPML1 is associated with a decrease in CMA as it was found to interact with Hsp70 and Hsp40, which are members of a chaperone complex required for protein transport into lysosomes during CMA [47]. It is hypothesized that TRPML1 may act as a docking site for intra lysosomal Hsc70 (ly-Hsc70), allowing it to more efficiently pull in substrates for CMA. It may also be possible that TRPML1 channel activity may be required for CMA [47].

#### 3.2.2. Nephropathic Cystinosis

Nephropathic cystinosis is a type of unusual autosomal recessive LSD which is caused by mutations in the CTNS gene (encodes a lysosomal cystine transporter, cystinosin) [48]; the treatment of cystinosis are currently based on the drug called cysteamine which reduces the cystine level [49]. This drug therapy reduces the disease progression rate; however, it does not cure other major problems caused by cystinosis like Fanconi’s syndrome and the final expansion of kidney damages. Hence, kidney cells have been shown to accumulate cystine in the lysosomes. Researchers have analyzed the disease pathogenesis in depth for a strong therapeutic approach by targeting the connection between altered mTOR signaling and autophagy in cystinosis. Certain expressional studies of clusterin (a protein involved in nephropathic cystinosis) partly cover the expression of apoptotic and autophagy proteins. 

Several detailed studies of autophagy in a cystinosis mouse model have been conducted; however, the autophagic flux and mTOR signaling were not well-studied. Furthermore, various studies have been conducted in yeast and protein interactions, where the role of cystinosin in mTOR regulation was analyzed. Their ERS1 protein fuses with the EGO (exit from growth arrest) which forms a complex (the yeast analog of the lysosome) in the vacuole. This analog of lysosomes regulates the mTOR signaling in yeast. Several cystinosis autophagic studies offer support for the dysfunction of autophagy in this LSD. Additionally, some studies reported that proximal tubular and fibroblastic cells encompass augmented autophagy of mitochondria in a cystinosis condition known as mitophagy. In cystinosis, there is an increase of the autophagosome marker (LC3-II and SQSTM1/p62) levels in cells which is reminiscent of autophagic flux dysfunction. However, CMA was impaired in cystinosin-deficient cells where LAMP-2A, the lysosomal receptor responsible for CMA, has dropped off expression and is abnormally localized [50,51]. The study shows that this impairment could not be treated by cysteamine therapy. Lysosomal dysfunction in cystinosis results in flawed autophagy-mediated clearance of impaired mitochondria. Impaired mitochondria encourage the production of oxidative stress that fuels tight junction ZO-1 phosphorylation mediated by Gα12/Src and promote a ZONAB signaling cascade of cell proliferation and transportation defects. The rectification of the lysosomal defect, the nullification of oxidative stress of mitochondria stress, and the disruption of ZONAB signaling salvage the function of epithelial cells from dysfunction [52].

The above-discussed link between the epithelial dysfunction and flawed lysosome–autophagy degradation pathways provides new therapeutic perspectives for lysosomal storage disorders. The increased apoptosis in renal proximal tubular epithelial cells showed a greater expression of a microtubule-associated protein, LC3-II/LC3-I, and substantially higher levels of autophagosomes in the variants of nephropathies. The autophagy inhibitor 3-methyl adenine rescued cystinotic cells from cell death. In certain studies of cystinotic cells, atypical mitochondrial function and increased levels of beclin-1 have been observed with a vast decrease in ATP generation in addition to an increase in reactive oxygen species. Additionally, a study has reported the precise inhibition of autophagy that results in significant diminution of apoptosis in nephropathic cystinosis [53]. Those studies provide data demonstrating atypical mitochondrial autophagy in nephropathic cystinosis, which may subsidize to renal Fanconi syndrome and progressive renal injury [54]. Still, more exploratory studies are needed to study the effects of modulators which target autophagy on the LSD phenotype in disease models. These studies will lead to the development of applicable treatments for numerous LSDs.

### 3.3. In Cancer: Biphasic Response

A complex association between autophagy and cancer may differ in different stages of the disease (Figure 2C). Accordingly, numerous studies have observed connections between the disease and the process in different stages and in different types. Reportedly, autophagy can be stimulated by some tumor suppressor genes, whereas the oncogenes are well-known to inhibit the autophagy, leading to an imbalanced homeostasis. The relationship between autophagy and cancer has long been proposed since 1990. According to distinct studies, the role of autophagy may fluctuate depending on the different stages of cancer development. Primarily in the initial stages of cancer, autophagy has a preventive effect: if there is a germinal of tumor outgrowth from the cancer cells, it might utilize autophagy for its self-cytoprotection [55]. On the other hand, the impaired autophagy may contribute to malignancy development [2,3,37,56]. 

Majorly, mutations in autophagy-involved genes and their genetic connectivity between the malignancy responses has been well-studied by researchers. For example, the mono-allelic deletion of BECN1 (a gene that encodes Beclin1, a mammalian orthologue of yeast Atg6/vacuolar protein sorting (Vps)-30) has been detected in many types of tumor specimens such as in human breast, ovarian, and prostate tumors [57]. Commonly, the Beclin1 protein interacts with the class III Phosphatidylinositol 3-kinase protein called Vps34 (also known as PIK3C3 in mammals) to form the Beclin1-Atg14-Vps34-Vps15 complex, which is important for the downstream autophagic proteins’ localization to the autophagosome formation site to induce autophagy [58,59]. In breast cancer cell-lines, the overexpression of Beclin 1 intensifies the rate of autophagy and hinders the cellular tumorigenesis [57]. The heterozygous deletion of Beclin 1 in mice resulted in diminished autophagy, and the outgrowth of tumors was found to be high as compared to the control mice [58,60]. Moreover, there are other autophagy-promoting components of the Beclin 1/Vps34 complex, namely UVRAG and Ambra1, which are known to be tumor suppressors [61,62]. 

A previous study in C. elegans showed that an additional protein called EI24/PIG8, whose human homolog was reported to be mutated in cancers (specifically breast cancer) [63], is a critical factor of autophagy [64]. Besides Beclin1 and EI24, the altered expression of several autophagy proteins such as Atg5 [65,66] and UVRAG [62] are also reported to be associated with various human cancers [67]. Kisen et al. 1993 [68] found that autophagic activity was shown to be lower in a primary cellular tumor in hepatocytes than in normal hepatocytes (in the liver of rat carcinogenesis models). Summarily the identification of the above-discussed genes required for autophagy provides the opening to use genetic approaches to explore the role of autophagy in cancer development.

#### 3.3.1. In Cancer Progression

Researchers have found that tumors can progress due to autophagy because during starvation, autophagy provides nourishment and thus allows the tumor to grow. Additionally, the study shows that during starvation or hypoxia, autophagy perks up p53-deficient cancer cells’ survival rate, also resulting in tumor progression. The metabolic stress-mediated accumulation of p62 was reported to be a significant phenotype of autophagy-defective tumor cells, which suggested the contribution of defective protein quality to tumorigenesis and indicated the turnover of p62 by tumor cells with the help of autophagy. Moreover, the failure of autophagy-defective tumor cells to eliminate p62 was enough for tumorigenesis [69]. In estimation, cancer-targeted therapies, including those that disrupt angiogenesis, inhibit the proteasome function or disturbance of signaling pathways, which can create other forms of cellular stress. Under these circumstances, autophagy plays a role to protect the cellular survival rate of tumors by providing them with nutrients and energy through the degradation of components of the cytoplasm for the recycling of amino acids and fatty acids for metabolism. Boya et al. 2005 [70] demonstrated on this condition the blunting of autophagy by impeding their early stage with 3MA treatment, followed by the inhibition of auto phagolysosome formation by hydroxychloroquine (HCQ) or bafilomycin A1 or the silencing of several genes such as Atg5, Beclin1, Atg10, or Atg12 by RNA interference, which has been shown to facilitate the death of starved cancer cells [70].

Guo et al. 2011 showed that even in the presence of copious nutrients, human cell lines have elevated basal levels of autophagy mutations in H-ras or K-ras; thus, it shows that autophagy provides metabolic substrates to maintain energy charge and nucleotide pools in Ras-driven lung cancer cells [71]. The repression of crucial autophagy proteins resulting in the inhibition of cell growth highlights the role of autophagy in tumor cell survival maintenance. In addition, it signifies that inhibiting autophagy in tumors that are captivated to autophagy, such as Ras-driven cancers, may be an effective treatment approach for cancer therapy. Similar studies on the Ras genes and their connection with autophagy show that some type of cancer cells, predominantly those porting an activated Ras oncogene, rely on autophagic activity for their endurance even in the lack of stress agents from environment [72,73]. To summarize, several researchers exemplified that in both in vivo and in vitro studies, autophagy supports the proliferation of cancer cells by adapting to their cellular stresses and maintaining their energy levels. In this way, the oncogenic mutated Ras expression amplified the autophagy, which may result in transformation and tumor growth [65,71,74,75].

Also, autophagy may protect cancer cells against the oxidative stress and DNA damage caused by chemotherapeutic agents by eliminating damaged macromolecules or organelles, allowing the sustained survival of cancer cells and thereby causing drug resistance. Furthermore, after cancer therapies such as radiotherapy or chemotherapy, constitutive upregulation of autophagy may favor a dormancy state in residual cancer cells, including CSCs (cancer stem cells), which may contribute to tumor recurrence and progression [55]. Senescence, a state of cell-cycle arrest which may serve as a tumor suppressor, has been suggested as a mechanism underlying tumor dormancy mediated by autophagy [76,77]. Studies have been demonstrated that autophagy arbitrates Ras-induced senescence and that the inhibition of autophagy holds up senescence in cancer cells [78,79]. The inhibition of autophagy has been shown to augment the efficacy of anticancer drugs [80].

#### 3.3.2. In Suppression

In contradiction with the above studies’ demonstrations of the role of autophagy in tumor progression, some studies have demonstrated the suppressive effect of autophagy on tumorigenesis on different models that are deficient for specific autophagy factors (Table 1). Takakura‘s [81] experiment on systemic mosaic Atg5 deletion or a liver-specific Atg7 deficiency mouse model with highly progressed liver benign tumors suggested that autophagy is needed for the suppression of spontaneous tumorigenesis in this model [81]. Similarly, an experiment on mice with Beclin1 heterozygous disruption by investigators Qu and Yue showed an amplified frequency of impulsive cancers [58,60]. Liang‘s study shows that normally a Beclin1 protein is expressed at an elevated level in breast epithelial cells, but in breast cancer cell lines (like MCF-7), it is expressed much less and the ectopic expression of Beclin1 triggers autophagy and inhibits proliferation and clonogenicity in xenograft mouse models [57]. Additionally, functional Beclin1 also inhibits proliferation of other tumor cell lines [37]. Besides Beclin1, several other components of the autophagic machinery were also found to suppress tumors, such as Atg4C and UVRAG. Experiments on mice with Atg4C deficiency showed an increased vulnerability to chemically-induced sarcomas [82].

The mono-allelic deletion of UVRAG (a positive regulator of the Beclin1-class III PI3K complex) and knockout of Bif-1 (an inducer of autophagy that interacts with Beclin1) were found to augment the growth of impulsive tumors [62,92]. Not only do mutations of the above autophagy gene promote tumorigenesis but autophagy is also regulated positively by the tumor suppressor genes and regulated negatively by the oncogenic signaling pathways. For example, Wang et al. in 2012 [93], found that when the Akt signaling pathway is activated, autophagy is often reduced. Thus, he concludes that Beclin1 is a protein target of Akt, and the phosphorylation of Beclin1 by Akt leads to the suppression of autophagy and links with oncogenesis, highlighting the Akt-mediated suppression of autophagy in the oncogenic process. Other tumor suppressors such as p53, TSC1, TSC2, and LKB1 trigger autophagy by mTOR inhibitory effects [94]. Therefore, autophagy controls oncogenes and tumor suppressor genes and modulates cellular processes by which tumor initiation and development are inclined [95,96], putting off necrosis and resulting in inflammation [97]. Impairment of the autophagic clearance due to chronic infection and exposure to toxins can cause chronic tissue damage and inflammation, which may contribute to cancer [98]. Hence these above-discussed studies provide an outline of the role of autophagy in developed cancers. It is also well-known from various studies that autophagy can have the opposite effect on the tumor development, enhancing it rather than suppressing it. These outcomes showed that there might be prospects for a beneficial treatment for cancer by inhibiting autophagy rather than stimulating it [99].

### 3.4. In Renal Disorders

From numerous literature surveys, it is well-known that autophagy has a significant role in the renal diseases (Figure 2C) [100]. Several studies have emphasized the defensive activity of autophagy in kidney disorder, typically in acute kidney injury, diabetic nephropathy, glomerulosclerosis, and cystic diseases. Different in vivo and in vitro models on renal injuries have been studied that show that an aggravated injury at the time of pharmacologic or genetic approaches induced autophagy inhibition. Consequently, autophagy in high levels was found to have promise as an approach for preventing or curing acute kidney injury. Meanwhile, the role of autophagy in chronic kidney disease is more complex and remains controversial compared to acute disease. Recent studies have been reported in the unilateral ureteral obstruction mouse model with the proximal tubule-specific deletion of autophagy-related genes that illustrate the contradictory results as compared to acute kidney diseases. The conditional deletion of autophagy related 7 (ATG7) in proximal tubules progressed renal fibrosis induced by unilateral ureteral obstruction [101]. Livingston et al. [102], studied the pharmacologic inhibition of autophagy with 3-methyladenine in the above mouse model. Contrariwise, similar experiments in mice with the conditional deletion of autophagy related 5 (ATG5) resulted in increased interstitial fibrosis, and rapamycin (a pharmacologic inducer of autophagy) reduced the unilateral ureteral obstruction stimulated renal fibrosis. This Atg5-mediated autophagy deficiency in proximal tubules promotes the cell cycle G2/M arrest and leads to renal fibrosis [103]. Emergent data has found that autophagy is obligatory for the regulation and biological functions of renal cells including proximal tubular cells and podocytes [104]. The above-discussed studies demonstrate a twofold role of autophagy in both mediating and modulating the diseases. In other words, autophagy is involved in both the regression and progression of acute and chronic kidney injury. More research is needed to identify the optimal context in which inducing or inhibiting autophagy might prevent progressive chronic kidney disease.

#### 3.4.1. In Glomerulosclerosis

Some clinical data has shown that sustained high levels of autophagy resulted in insignificant changes in the patients of renal disease. On the other hand, in the case of diminished levels of autophagy, the progression of Focal Segmental Glomerulosclerosis (FSGS) has been observed and associated to podocytopathies [105,106,107]. Several studies reported that the inhibition of autophagy might promote the podocyte apoptosis [108,109]. For example, Zeng et al. 2014 [110] reported that the high frequency of autophagy inhibition might contribute to the apoptosis of podocytes. This above-study displays that podocyte autophagic activity plays a critical defensive role in renal injuries and that sustaining the autophagic activity of podocytes signifies a conceivable beneficial therapeutic approach for the regulation of podocytopathies [110]. In 2013, Liebau MC et al. [111] reported that podocyte-specific conditional ATG5 knockout on mice caused albuminuria and glomerulosclerosis. In addition to the above-discussed report by the group of Zeng, the histologic and clinical study on FSGS showed the loss-of-function of ATG5 or ATG7 mutations in mice [112]. As a result, the role of autophagy in cyto-protection involves regulating the progression of podocytopathies.

#### 3.4.2. In Fibrosis

In vivo studies on autophagy infers that it has a dual role such as in the upregulation and downregulation of autophagy in fibrogenesis. Kawakami et al. [113], reported that TGF-β1 functions both as an inducer of collagen synthesis and as an inducer of autophagy and subsequent collagen degradation. Interestingly, through the pathways of PI3K/Akt, the TGF-β protein can trigger the mTOR pathway, and consequently, mTOR can exert both stimulatory and inhibitory effects on autophagy. Studies on certain pharmacological molecules such as metformin and their roles in inducing AMPK have shown them to be a promising beneficial target in reducing renal fibrosis and its therapeutic interventions through regulating autophagy [114].

#### 3.4.3. In Diabetic Nephropathy (DN)

In both types of diabetes, enhanced mTORC1 and rapamycin were reported to stop the progression of DN [115,116]. Moreover, obesity-mediated autophagy is also connected to the hyper activation of mTORC1 [117]. Huber et al. [100] reported the increased mTORC1 in human and animal DN podocytes [100]. The diminished autophagy activity was observed in the kidneys of streptozotocin-induced diabetic mice, high-fat-diet-induced obese mice, and wistar fatty rats [118]. The pathway of AMPK is probably repressed during the development of DN [36]. In conclusion, reports have collectively shown that the hyper activation of mTORC1 may be significant for the progression or onset of DN. Similarly, the stimulation of autophagy via starvation or the restriction of calories and the activation of AMPK can likely be a target for refurbishing the process of homeostasis in DN.

#### 3.4.4. In Transplantation/Renal Ischemic Injury (IR)

Numerous studies reported that autophagy influences renal IR injury [119,120,121,122,123]. Similar to other renal disorders, studies reported that induced and stimulated autophagy during renal IR also served to express the protective defense mechanism for maintaining homeostasis throughout hypoxic starvation in ischemia and to counteract the oxidative damaged proteins and organelles during reperfusion [119,124,125]. In ATG5- and ATG7-(proximal tubule-specific) knockout mice, autophagy presented a protective role in the condition of renal IR [126,127]. The consequences of the modulation of autophagy may depend on the ischemic length stretch in the animal model. Longer durations of ischemia could provoke kidney cells to undergo autophagy-dependent cell death or autophagy impairment associated with autophagosome accumulation post-reperfusion [128].

#### 3.4.5. In Cystic Diseases

Autosomal dominant kidney disorder (ADPKD) is a well-known cystic disease, in which an activated mTOR pathway is observed in both in vivo and in vitro models. MEK/ERK pathway activation was also observed in ADPKD. Cystic disease is a chronic kidney disorder, and reports have claimed that enhanced autophagy may play a protective role in this disease. Autophagosomes and LC3-II (protein involved in autophagy) exist in the tubular cyst-lining cells in the Congenital Polycystic Disease (CPD) kidneys of mice but are found to have insignificant or no increase in autophagic flux. Mutated PC1 (Polycystin-1) mouse kidney cells showed totally negative autophagy induction in response to glucose withdrawal as well as high levels of apoptosis. Treatment with rapamycin-like compounds led to a higher cell survival. Precisely, autophagy in ADPKD is impaired, and the induction of autophagy may have a promising role in therapeutic development for ADPKD [129].

### 3.5. In Inflammatory and Infectious Disease

Recent studies have shown that autophagy has an association with several inflammatory and infectious diseases (Figure 2D), including Crohn’s disease, pulmonary hypertension, chronic obstructive pulmonary diseases, and some systematic inflammatory diseases. Reports from several different studies have confirmed a strong relationship between autophagy and infectious diseases, and this connection is beneficial for treatment strategies. Thus, it overlays a concept that the modulation of autophagy might lead to therapeutic interventions for diseases associated with inflammation. Here we discuss the various roles of autophagy in inflammation as well as inflammatory diseases. The interactions of autophagy with inflammatory cytokines or chemokines was reviewed by Harris in 2011 [130]. Autophagy can affect the secretion of cytokines by affecting the secretion of Th1 cytokines, IFN-γ, TNF-α, IL-1, IL-2, IL-6, TGF-β, and MCP-1 and Th2 cytokines, IL-4, IL-10, and IL-13, as well as other cytokines, IL-1β, IL-18, IFN-a, IFN-β, and IL-8 IL-1β production to the base. In macrophages, the presence of Atg proteins (vital for autophagy process) has been reported and a few knockout studies represent that without Atg16L1 or Atg7, there would be an increase in interleukin (IL)-1β and IL-18 productions in reaction to inflammation induction by receptor-mediated signaling known as TLR (Tor like receptor). Moreover, the pathway of TLR could also augment the phagosomes and auto-phagolysosome formation by the aid of above-discussed proteins of Atg5 and 7, encoded by ATG5 and ATG7 respectively. The increase in auto-phagolysosomes results in the increased removal of debris in monocytes/macrophages [130,131]. A study on the knockout mice model of an autophagy protein called Atg5 revealed that macrophages and neutrophils are highly prone to infection in the circumstance of autophagy defects [132]. Presently, conclusions from various studies have shown that autophagy is involved in enhancing the caspase-independent cellular death in stimulated macrophages. Lai et al. 2015 [133] showed that fatality of stimulated macrophages might also be promising in regulating the rate of inflammation. A group of researchers [134] reported that certain molecules which are adhesive in nature can provoke the autophagy-dependent and caspase-independent cellular death in neutrophils. The molecules were categorized by their cumbersome cytoplasmic vacuolization seen in septic shock, cystic fibrosis, rheumatoid arthritis, and several skin diseases [135]. The above principle underscores that autophagy stimulation in cells is a wide-ranging occurrence following a neutrophilic inflammation reaction. Studies on the inhibition of neutrophil extracellular traps cell death (NETosis) reported by Tang et al. in 2015 [136] has shown that autophagy inhibits NETosis through intracellular chromatin de-condensation prevention, resulting in apoptotic cell death.

#### 3.5.1. In Infectious Disease—HIV

Currently around 40 million people are living with HIV around the worldwide, in addition to 30 million more that have been killed by this virus. Modern treatment regimens result in HIV suppression and immune recovery. However the spread and the prevalence of this virus has been increasing day by day as discussed by Joska et al., 2010 [137] and others, indicating the necessity for alternative therapy or preventive approaches. Acquired immunodeficiency syndrome (AIDS) is a highly pandemic disease among infectious diseases. Designing the ideal vaccine or therapeutics against this virus has been difficult due to its high genetic heterogeneity as a result of the high replication and mutation rates leading to the production of many genetic variants and recombinants. The high genetic diversity of this virus has increased the concern for developing countries like India, where it has generated various recombinants such as A/C, A/E, and B/C [138]. Critically, genetic variations in the viral genes like Tat and Vif result in differential viral activities, such as 1. varying levels of Tat interaction with the transactivation response RNA (TAR) element [139] and 2. Vif gene-induced differential APOBEC3 degradation [140], which results in a drastic increase of viral gene expression [141]. Among those, HIV-Tat plays a major role in regulating autophagy by affecting IFN-γ signaling through the suppression of STAT1 phosphorylation and consequently inhibiting major histocompatibility complex class-II antigen expressions. Also, HIV 1-Tat suppresses the induction of autophagy-associated genes and inhibits the formation of autophagosomes [142]. Distress of autophagy by HIV-1 would harm the active repression of attacking pathogens, thereby providing a favorable environment for opportunistic microbes in HIV-infected individuals [143]. It is noteworthy to report that autophagy plays a complex role in HIV infection, and this has been validated by signifying the critical functional role of autophagy during HIV infections on different phases. HIV requires autophagy for accomplishing its replication steps to create replicas, although the process of autophagy has manifold strategies to evade the recognition and degradation of the newly synthesized viral particles. In HIV infections, the dependency of autophagy genes in (HIV) replication has been testified by the screening of wide-ranging RNA interferences (RNAi). Brass et al. 2008 [144,145] showed that in HIV infection mechanisms, certain autophagy genes, namely GABARAPL2, ATG12, ATG7, and ATG16L2, played a vital role. RNAi studies and several pharmacogenomics approaches confirmed the part of autophagy as pro-viral in both macrophages and T cells [145,146]. A direct connection between the HIV proteins and the proteins involved in autophagy machinery was identified, specifically between the autophagosome protein LC3 and HIV GAG precursor. Notably, this association is required for the proper processing of GAG, indicating that the autophagosome may provide membrane support for viral replication [147]. Targeting these key autophagy proteins or viral proteins by utilizing novel RNAi technology, such as CRISPR Cas9, will help in perturbing the viral infectivity.

#### 3.5.2. Autophagy in HIV and HIV-Associated Neurological Disorders (HAND)

Exploring the molecular mechanisms of HIV-1 proteins such as Tat, gp120, and Nef (found to be involved in different phases of autophagy, i.e., initiation and maturation) linked to autophagy is also significant because it plays dual roles in HIV-1 replication and HIV-1 disease progression [148,149,150]. Thus, this section reviews the interactions between HIV-1 and autophagy [148,151,152]. 

Numerous studies have been reported connecting the autophagy mechanism with HIV-1 proteins. Interestingly, HIV-1 has evolved so that it can counteract the degradation effect of autophagy. HIV-Tat has been found to cause a decrease in Beclin-1, LC3II, and p62 levels in neurons, suggesting that Tat may induce autophagic degradation by inducing the fusion of autophagosomes and lysosomes [153,154]. Tat suppresses autophagy in macrophages [153,155]. Evidently, neuronal autophagy levels are altered by Tat by inhibiting the fusion of autophagosome with the lysosome [153]. HIV-1 Nef interacts with Beclin-1 [147] and blocks the autophagosome maturation [156,157], causing a decrease in autophagic influx in infected cells, thereby allowing for their replication and avoiding their fate of being degraded [147]. In HIV gp120 transgenic mice, BECN1 levels are altered and ASPP2 knockdown reduced autophagy along with apoptosis induced by gp120 [158,159]. HIV-1 Env has been shown to activate mTOR in dendritic cells [160,161], leading to the exhaustion of autophagy and transfer of the infection into CD4+ T cells. These findings represent one of the pathological mechanisms that can be utilized as a potential therapeutic target for HIV-1 [157,162]. HIV-1 Env causes autophagy-dependent cell death via binding to CXCR4, a cell surface receptor. Thus, autophagy is important for Env-induced apoptosis through CXCR4 [163]. In CXCR4- or CCR5-tropic gp120 exposed neuronal cells, an increased amount of autophagosomes was reported [164]. 

Similarly, a clinical study on HIV-associated encephalitis patients showed that autophagy-related proteins such as Beclin-1, Atg5, Atg7, and LC3II are found to be significantly increased in the brains of the affected individuals [165]. However, HIV-1 patients without encephalitis have no observed changes in the level of autophagy [164]. The studies discussed above, as well as several others, suggest that autophagy is crucial for neuronal homeostasis [166]. Neuroglial toxicity has been reported to result from the suppression of autophagy via X4 HIV-1, whereas R5 HIV-1 only causes toxicity in neurons [167]. Reported recent studies have demonstrated that early HIV proteins are not affected by combined antiretroviral therapy and that they can deregulate the ER stress pathways and autophagy activity, playing a crucial role in the development of HAND [168]. During HIV infection, autophagy is repressed in infected CD4 T cells by Env, which supports the replication of viruses, while HIV-1 Tat is degraded by autophagy, indicating the anti-HIV effect of autophagy. 

Meanwhile, in infected monocytes and macrophages, autophagy is provoked by Env [169]. In addition to Env, gp120 binding to the NMDA receptor in cardiomyocytes induces autophagy [170]. Tat protein blocks autophagy through Src-Akt and STAT3 signaling [155]. In Human macrophages, Tat suppressed IFN-γ-induced autophagy [155]. In T cells, Tat is selectively degraded by autophagy [171]. In Glial cells, Tat induces autophagy through the enhancement of BAG3 protein level [172]. In neurons, Tat induces the autophagic degradation by promoting the fusion process [153]. In macrophages, Gag-derived proteins co-localized and interacted with the autophagy factor LC3, and autophagy promoted productive Gag processing while the Nef inhibited autophagy maturation through interactions with Beclin-1 [147]. In infected macrophages, Nef modulates TFEB localization and alters autophagy degradation [173]. In human astrocytes, Nef mimics Baf A1 and blocks the formation of autophagolysosomes [174]. In HeLa cells/hepatocarcinoma, Nef induces autophagy through an IRGM dependent pathway [175]. In bone marrow mesenchymal stem cells, Nef treatment causes the early inhibition of autophagy, thus inducing MSC senescence [151,176].

#### 3.5.3. Endoplasmic Reticulum (ER) Stress-Mediated Autophagy

The intracellular accumulation of misfolded proteins in the ER causes stress and triggers the unfolded protein response (UPR), which induces the expression of chaperones and proteins involved in the recovery process [177]. Increased levels of autophagy have also been found to be related with ER stress pathway and several inflammatory factors such as TNF, IL-1β, IL-6, and CCL2 [178,179]. The signaling pathways of IRE1α, PERK, ATF6, and Ca2+ activates ER stress-induced autophagy [180,181]. ER stress has been reported in neurodegeneration and found to be stimulated by the accumulation of misfolded proteins related to various neuro-disorders [182,183,184]. However, the receptor-mediated selective ER-autophagy degrades the ER by Atg40, which is probably the functional counterpart of FAM134B. Atg40/FAM134B is an autophagy receptor for the ER in mammals that has been implicated in sensory neuropathy [185]. ER calcium store depletion is also a cause of ER stress via the ITPR and RYR channels in HIV-associated neuro-disorder [186,187]. The pathogenic accumulation of misfolded proteins leading to the activation of the UPR [188] results in the upregulation of mediators such as HSPA5 and the phosphorylated forms of EIF2AK3 and EIF2S1 (indicators of ER stress) in various neurodegenerative disorders [189,190,191,192,193,194]. Several studies suggest that autophagy serves as an essential machinery to regulate the homeostasis by UPR [180,195,196]. The effect of ER stress resulted in the following activity: UPR downstream mediators of EIF2AK3 such as ATF4 and DDIT3 were also reported to guide the induction of autophagy gene transcription such as with BECN1, MAP1LC3B, ATG5, ATG7, and ATG12 [197]. PARK2 when transfected in dopaminergic neuroblastoma-derived SH-SY5Y cells showed more resistance to apoptosis mediated by ER stress [198]. XBP1s, an UPR mediator, can reduce the expression levels of MAP1LC3B-II in fALS and thus hinder autophagy activity. The ubiquitin ligase PARK2 is also important for the relationship between ER stress and autophagy. The increased secretion of SNCA from the brain into the blood has been observed during diminished interactions between PARK2 and BECN1 in mouse models of PD. The elevated level of MAP1LC3B (autophagy initiator) in the brain of AD patients has been found in the phosphorylated-EIF2AK3-positive neurons [199]. The loss of BECN1 (autophagy initiator) rises the deposition of Aβ intracellularly in neurons, and in microglial cells it decreases the Aβ clearances by phagocytosis. The dysregulation of autophagy lead to glial cell-mediated neuroinflammation [200,201]. Aggregated Aβ damages lysosome in the absence of BECN1, which releases the lysosomal protease CTSB and activates the IL1B pathway, resulting in a microglia-dependent inflammatory response [202]. Autophagy affects microglial phagocytosis of apoptotic cells, amyloid-β, synaptic material, and myelin debris and regulates the progression of age-associated neurodegenerative diseases [200,203]. Although, both in vitro and in vivo approaches showed the molecular mechanisms that determine the outcome of UPR and autophagy activation to improve therapies for cancer and neurological diseases treatment [204,205]. Moreover, Tat is known to be capable of activating ER stress pathways in various types of cells in the brain. Tat-induced apoptosis of human brain microvascular endothelial cells involves ER stress activation and mitochondrial dysfunction and upregulates the ER stress markers including HSPA5, ATF6, and phosphorylated EIF2S1 in both cortical neurons and astrocytes [206,207,208]. HIV gp120 and Nef have the ability to affect autophagy levels by targeting the different phases in the autophagy machinery. [171]. Some pathogenic proteins such as HIV Tat can escape autophagic clearance via interference with the endolysosomal pH, inactivation of endolysosomal enzymes, and inhibition of autophagy.

#### 3.5.4. Autophagy and Drug Abuse-Mediated Cytotoxicity

The machinery of autophagy is known to be affected by the drug toxicity mediated by different drugs used for drugs abuse. While, their mechanism varies in respect to different cell types and different drugs administered. Studies have been demonstrated that autophagy protects the cells from drug-induced toxicity, and cells are vulnerable to apoptosis as an effect of the obstruction in autophagy. However, the observation of cell death has been reported during drug administration followed by excessive autophagy. The excessive autophagy-mediated cell death can be reversed by blocking the autophagy pathway. The most common known drugs which are abused and extremely affects autophagy are cocaine, METH, morphine, and alcohol. The abuse of prohibited drugs is understood to be one of the vital risk factors for the causes and progression of HIV-1. A study showed the induction of autophagy on the exposure to cocaine in primary rat microglial cells and BV-2 cells by the induction of autophagy-signature proteins such as BECN1/Beclin 1, ATG5, and MAP1LC3B in dose- and time-dependent manners. During drug-induced toxicity, autophagy activity has been proved to be altered in various cell types. Here, we review the current literature on the interaction between autophagy, HIV-1, and drug abuse and discuss the complex role of autophagy during HIV-1 pathogenesis in the co-exposure to illicit drugs.

Autophagy mediated by the exposure of methamphetamine (METH) has been reported in neurons [209]. METH neurotoxicity results from the induction of a specific cellular pathway that is activated when DA cannot be effectively sequestered in synaptic vesicles, thereby producing oxyradical stress, autophagy, and neurite degeneration [210]. METH promoted the formation of autophagic granules, particularly in neuronal varicosities and, ultimately, within the cell bodies of dopaminergic neurons. In mouse’s primary midbrain neuronal cells, METH-induced neurodegeneration, and the autophagic vacuoles were observed. METH treatment in PC12 cells showed the occurrence of autophagosomes and increased levels of Beclin-1 and LC3II [211]. An in vivo study on METH-induced rats showed the upregulation of ubiquitinated protein levels, PKCδ cleavage, and LC3II. Several studies confirmed the protective role of autophagy against the cell apoptosis induced by METH [212]. However, a few studies reported that autophagy has been linked to neurotoxicity induced by the exposure of METH in certain cells. In a therapeutic study of neuroblastoma, the study reported by inhibiting the autophagy via mTOR activation to understand the role of autophagy in the treatment of METH-induced cell death. This above mTOR activated autophagy inhibition has been shown to reduce cell death in human neuroblastoma SK-N-SH cells [213]. The above-discussed studies suggest the involvement of autophagy in METH-induced neurotoxicity. HIV-positive individuals may have an increased sensitivity to methamphetamine, leading to a high methamphetamine abuse potential in this population [214]. Studies on antioxidants which diminish the level of autophagy and METH-induced cell death showed the correlation between autophagy and apoptosis under stress condition, and this can be regulated by the Beclin-1: Bcl-2 complex as reported [211]. Autophagy has been implicated as one of the possible mechanisms that contribute to morphine-mediated cell death. Preceding morphine itself, morphinone, one of the active metabolites of morphine, has been found to induce non-apoptotic cell death in human promyelocytic leukemia cells [215].

A study has been reported by revealing the role of autophagy in drug toxicity, such as the inhibition of autophagy pathway reduced the morphinone-induced cytotoxicity leading to caspase 8 or 9 dependent cell death. This displayed the involvement of autophagy in the process of caspase 8 or 9 dependent cell death mediated by morphinone. However, the mechanism that facilitates autophagy to associate with morphinone-induced cytotoxicity is still uncertain. In human neuroblastoma SHY-5Y cells, the drug morphine encourages autophagy by intermediating with the opioid receptor-dependent pathway, along with Beclin-1 and Atg5 [216]. A study showed the importance of autophagy in morphine mediated cell death by discussing that autophagy inhibition resulted in the worsening of the degree of cell death induced by morphine. Similarly, the activation of autophagy in neuronal cells led to a diminished level of cell death and reduced the inflammation of the hippocampus which represents the morphine-induced memory impairment [217]. Chronic morphine use, or the prolonged use of morphine, was connected to mitochondrial dysfunction, and the mitochondrial DNA copy number reduction has been reported to be aided by autophagy and has reported that the autophagy activation by lipopolysaccharide was induced by morphine [218,219]. Though, the cocaine-induced autophagy is less reported, studies have been in quest of understanding the interlink of cocaine cytotoxicity and autophagy. The administration of cocaine in various regions of the primary neuronal cell model showed a higher level of autophagy marker expression and autophagosome formation. The prolonged exposure of cocaine resulted in higher cell death, which can be treated by small molecule mediated inhibition of the autophagy pathway. A complete reverse in cell death has not been achieved by the above case, suggesting that several other mechanisms are also responsible for cell toxicity and cell death via chronic exposure of cocaine along with autophagy [220]. The autophagy process has also shown to be reduced in different cell lines such as human monocytic U937, CD4 Jurkat, MCF-7 cells, cardiomyocytes, and cortical neuroepithelial progenitors and observed to inhibit the AMP-activated protein kinase (AMPK), an upstream regulator of autophagy when exposed to alcohol, resulting in the suppression of the downstream autophagy signaling pathway via mTOR [221,222,223]. Furthermore, the vesicular movement in hepatocyte is disrupted by alcohol; this results in the inhibition of the formation of autophagosome [224]. Failure in the clearance of cellular organelles damaged by alcohol, such as mitochondria, has been reported. Yet, several studies have proposed that alcohol consumption initiates autophagy induction.

## 4. Autophagy and Therapy

Recently, researchers have found that directing autophagy-like processes might be a feasible therapeutic approach in fighting diseases, specifically cancer and neurological disorders. Different approaches have been found for the treatment of various diseases by targeting autophagy. In cancer, one method of treatment is to induce autophagy and enhance its tumor suppression attributes. Autophagy can also be inhibited to treat cancer based on the concept that autophagy is a protein degradation system used to maintain homeostasis, and some studies suggested that the inhibition of autophagy often leads to apoptosis. However, the inhibition of autophagy is riskier as it may lead to the survival of the cells instead of the targeted cell death [225]. So far, various pharmacological studies have been examined and tested as autophagy-induced therapies in diseases. These therapies indicate that autophagy increases in a dose-dependent manner. This dose dependency of autophagy is directly related to the growth of cancer cells in a dose-dependent manner as well. In conclusion, many cancer researchers have undergone various studies of therapeutic approaches to encourage autophagy in cancer, one of which includes the inhibition of the protein pathways directly known to induce autophagy that may serve as an anticancer therapy.

## 5. Discussion

Most studies on autophagy and diseases are rationalized by the specific objective of identifying small molecules or pharmacologic agents that have “off-target” properties beyond prompting or hindering the autophagy. Some groups experimented on the genetic knockout models of autophagy-related proteins such as Atg8, Gabarapl2, Atg12, Atg7, and Atg16l2 to identify their helpful or adverse effects in certain diseases. Future potential studies could be done on developing drugs that are more specific in targeting autophagy. More exploration on CRISPR knockout in in vitro models and in vivo models with the inducible modulation of genes involved in autophagy during specific disorder or in various diseases are mandatory to define whether focusing on autophagy would be a feasible approach in the future, in which autophagy-modifying medications may be used to impede or treat diseases. Overall, within the past few years, autophagy has been hypothesized and has emerged as a new and potent modifier of disease progression or suppression rates. We have discussed the recent significant findings on the role of autophagy in various diseases and its implications in novel therapeutic approaches for the betterment of humans. Moreover, an improved understanding of the relevance of autophagy in the different stages of various disease progression is obligatory. Additionally, the revolutionary approaches should be engaged to recognize discrete aspects of autophagy in diseases that can be overwhelmed, targeted, and used for the progress of a practicable treatment approach to cure various diseases efficiently.

## Figures and Tables

**Table 1:** The phenotypes and diseases associated with an alteration of the autophagy genes.

Genotype of the Autophagy-Related Genes	Phenotype	Relevant Diseases	Reference
Atg5 deletion, liver-specific Atg7 deficiency and Atg4C deficiency	Mice develop benign tumors in the liver.	Liver cancer	[81]
Atg7 mutation	Mice show impaired glucose tolerance and a decreased level of serum insulin: protective autophagy.	Diabetes	[83]
Atg5 deficiency	Mice show cardiac hypertrophy and contractile dysfunction.	Cardiomyopathy	[84]
Beclin1 deletion	Mice show an increased frequency of spontaneous tumors: protective autophagy.	Breast, ovarian, and prostate cancer	[58,60,84]
Beclin1 overexpression	Decreases in MCF-7, cellular proliferation, in vitro clonogenicity, and tumorigenesis in nude mice.	Breast cancer	[84]
LAMP2 deficiency	Ultrastructural defects in cardiac myocytes and severely reduced cardiac contractibility.	Danon disease	[85]
Ambra1 deficiency	Severe neural tube defects in mice.	Neurodegenerative disease	[61]
Beclin 1 deficiency	Accelerated amyloid-b accumulation: protective autophagy.	Alzheimer’s disease	[86]
Bec-1, Ce-Atg7, or Ce-Atg18 knockdown	Increase in aggregate formation and toxicity of PolyQ expansion proteins in Caenorhabditis elegans.	Huntington’s disease	[31]
Beclin1 overexpression	Reduced accumulation of a-syn and the associated neuronal pathology in mouse.	Parkinson’s disease	[87]
cathepsin D^−/−^ (all tissues)	Neuronal ceroid lipofuscinosis with autophagic vacuolization and LC3-I to LC3-II conversion. Bax knockout reduced enhanced apoptosis but not autophagic degeneration and neuronal loss.	Neurodegenerative disease	[88,89]
cathepsin B^−/−^L^−/−^ (all tissues)	Severe brain atrophy with enhanced apoptosis, autophagic vacuolization, and LC3-I to LC3-II conversion.	Neurodegenerative disease	[88,89,90]
cln3 (all tissues)	Juvenile neuronal ceroid lipofuscinosis, with autophagic vacuolization and LC3-I to LC3-II conversion.	Neurodegenerative disease	[91]
ambra1^−/−^ (all tissues)	Decreased autophagy, increased apoptosis, and increased cell proliferation in fetal brain. Neural tube defects and embryonic death.	Neurodegenerative disease	[61]
